# Electrical, Thermo-Electrical, and Electromagnetic Behaviour of Epoxy Composites Reinforced with Graphene Nanoplatelets with Different Average Surface Area

**DOI:** 10.3390/polym14245520

**Published:** 2022-12-16

**Authors:** Ignacio Collado, Alberto Jiménez-Suárez, Rocío Moriche, Gilberto Del Rosario, Silvia Gonzalez Prolongo

**Affiliations:** 1 Materials Science and Engineering Area, Escuela Superior de Ciencias Experimentales y Tecnología, University Rey Juan Carlos, Tulipán Street, 28933 Móstoles, Spain; 2 Department of Condensed Matter Physics, University of Sevilla, Apartado 1065, 41080 Sevilla, Spain; 3 Technological Support Center, University Rey Juan Carlos, Tulipán Street, 28933 Móstoles, Spain

**Keywords:** nanocomposite, graphene nanoplatelets, conducted EMI shielding, thermo-electrical behaviour

## Abstract

The influence of the average surface area of different graphene nanoplatelets (GNP) on the thermo-electrical behaviour, associated with Joule heating, and the attenuation of electromagnetic signals of epoxy composites has been studied, analysing the effect of the morphology obtained as a function of the dispersion time by ultrasonication and the GNP content added. Gravity moulding was used as the first stage in the scaling-up, oriented to the industrial manufacture of multilayer coatings, observing a preferential self-orientation of nanoparticles and, in several conditions, a self-stratification too. The increase of sonication time during the GNP dispersion provides a decrease in the electrical conductivity, due to the GNP fragmentation. Instead, the thermal conductivity is enhanced due to the higher homogeneous distribution of GNPs into the epoxy matrix. Finally, the lower surface area of GNPs reduces the thermal and electrical conductivity due to a greater separation between nanosheets. Regarding the study of the attenuation of electromagnetic waves, it has been discovered that in the frequency range from 100 Hz to 20 MHz, this attenuation is independent of the direction of analysis, the type of graphene, the sonication time, and the state of dispersion of the nano-reinforcement in the matrix. Furthermore, it has also been observed that the conservation of the constant shielding values for the three types of GNPs are in a range of average frequencies between 0.3 and 3 MHz.

## 1. Introduction

Nanoreinforced polymer composites are the most widely used nanoreinforced matrices because they are the simplest to process, obtaining good dispersions of the nanofillers compared with other composite materials [1]; they are also lightweight and versatile [2].

The applications of polymer nanocomposites are promising and numerous. Their use is currently mainly focused on research, but there are already numerous industrial uses, which will expand over the years [3], in particular, those related to the electronics sector [4], renewable energies [5], and transport, especially aeronautics [6]. Among the most common applications closely related to nanocomposites are the monitoring of structural health through electrical resistance changes [7], electromagnetic shielding in the required specific bands [8], anti- and de-icing devices [9], and the self-repair, adhesive repair [10], or adhesive curing [11] by Joule or electromagnetic heating in nanoreinforced networks that exceed the percolation threshold [12], among others.

The nanoreinforcements used in this research are graphene nanoplatelets (GNPs). Graphene, which is basically a sheet of hexagonal carbon structures (of sp^2^ bonds) shows much more interesting properties than graphite, due to its higher charge carrier mobility (10^5^ cm^2^/V∙s), larger theoretical specific surface area (around 2630 m^2^/g), its high Young’s modulus (1 TPa), its high thermal conductivity (4000 W/mk), and its control of optical transparency [13,14]. Graphitic nanoplatelets show an intermediate behaviour between graphene nanosheets, which are expensive and difficult to disperse and stretch, and graphite. In order to reach the optimum behaviour of the nanocomposites, the GNPs must be well dispersed and even exfoliated.

Regarding the processing of these materials, it should be noted that the greatest difficulty lies in obtaining a good dispersion of the nanoreinforcements. This difficulty is justified by the great surface area associated with the nanoparticles, which produces a great chemical and physical interaction, enhancing the formation of agglomerates [15]. In the case of epoxy nanocomposites, the most common manufacturing technology is the named in situ polymerisation [16], which implies the dispersion of the nanoreinforcement into the non-reacted monomers. In this work, sonication has been used as the dispersion technology. Sonication has been demonstrated as an efficient method to weaken interlayer forces [17], enhancing both the dispersion and the exfoliation of GNPs. This method consists of the generation of sound waves that propagate alternating high and low pressure cycles, with frequency-dependent velocities.

Time and intensity of the sonication dispersion are the most influential parameters in the morphology of GNP nanocomposites, and therefore in their final properties. Sonication intensity refers to the probability of cavitation events per unit volume occurring, while sonication time is defined as the total energy input to disperse [18]. These two parameters are what control the evolution of nanoreinforcement in the matrix. Some researchers [19] have suggested the existence of two well-defined stages during the defect formation process: the first stage, which is called the amorphization path, or low disorder or nanocrystalline regime, and the second stage, which is called the high disorder or amorphous carbon regime. The existence of these two stages allows us to predict that there will be a maximum sonication time for which the functional properties of our nanocomposite begin to be lost, because the damage generated begins to exceed the benefits provided by the dispersion.

Both intense energy and prolonged time tend to cause localised damage that can degrade the graphene structure by introducing structural and topological defects such as vacancies, dots, edges, and dislocations in the sp^2^ C-C lattice of graphitic walls [20]. In extreme cases [21], uncontrolled sonication leads to a high sp^3^ content, corresponding with amorphous carbon, and the scissors effect, i.e., a shortening of the aspect ratio. These phenomena drastically affect the unique mechanical and thermal properties of graphene nanoreinforcement, as well as its ability to conduct electrical charges.

If GNPs have a good dispersion into the matrix, they produce improvements in diverse properties, including mechanical, electrical, and thermal properties. The improvement in mechanical properties has been corroborated in numerous studies [22,23,24], in which different techniques are used, such as nanoindentation, tensile tests, Izod and Charpy impacts, bending tests, and load–retention–unload cycles, to measure various mechanical properties. These tests have shown that an increase in the percentage of reinforcement implies an improvement in the mechanical properties, as long as the processing method has been carried out correctly [25]. Apart from obtaining improvements in mechanical properties, polymer nanocomposites can undergo a transition from insulator to electrical conductor, which is justified by the formation of a path of electrically conductive charges through the insulating polymeric matrix [26]. This network is sometimes not sufficiently dense or not well dispersed, causing the existence of unrelated finite groups that end up generating so that the network is not obtained. However, it is known that there is a reinforcement fraction, called the percolation threshold, for which there is a good dispersion. Thus, the electrical conductivity grows exponentially for a not very wide variation in the near fraction, until reaching a limit [27].

The mechanism is represented by the percolation theory [28], which exposes a phenomenological equation that allows explaining the conductivity of a system near the conductor–insulator transition zone. Aaron Krieg [29] confirmed a loss of electrical resistance with a logarithmic trend in polymer nanocomposites reinforced with graphene, obtaining a percolation threshold of 12% by weight, which is equivalent to 7% by volume. However, the percolation threshold values are not universally valid, depending on the type and geometry of conductor nanofiller and even the insolating matrix. One of the lowest values obtained for the percolation is about 0.25% of chemically modified graphene platelets by volume, obtained by Zaman and colleagues [30].

The conductivity is also related to the nanofiller content, its dispersion, and therefore to the processing. Although there are mathematical models for its prediction [31,32], complex behaviours are being published depending on the appearance of aggregate reinforcement fractions. In the case of sonication dispersion, higher conductivities are measured for long sonication times (3 h) at high GNP contents [33] because the viscosity of the medium increases, reducing the cavitation forces and therefore reducing the significant breakage of the nanoplatelets.

The thermal conductivity of thermosetting polymers is usually limited because the phonon mean free path is extremely small in amorphous polymers, so the phonon scattering is enormous [34]. In this sense, the additions of graphitic nanofillers, such as GNP, are considered as promising candidates to be added due to their very high intrinsic thermal conductivity [35]. Although thermal conductivity does not have a percolation threshold [36], this parameter is also related to the dispersion of the nanoreinforcements, and therefore to the processing of the nanocomposite. Higher thermal conductivities are obtained for long sonication times when a certain weight percentage is exceeded [33], as occurred with the electrical conductivity. The increase of the aspect ratio during a correct sonication induces an improvement in the thermal performance [35,37].

Electrical conductivity induces the occurrence of the thermoelectric phenomenon, which appears when a voltage difference is applied to a conductive material. The electric current flows through it, and the electrical energy is dissipated as heat to overcome the resistance linked to the friction between the electrons and the atoms, molecules, or chain segments of the material. This heating is known as the Joule effect. The amount of heat produced is proportional to the square of the current, the resistance of the circuit, and the time during which the current flows through the circuit, although modified potential [38] and exponential trends have been observed in some polymer composites with carbon nanoreinforcements.

Finally, it is important to highlight that polymer nanocomposites present a considerable interest due to their ability to absorb electromagnetic microwaves. This makes them candidates to improve the compatibility and effectiveness of electromagnetic shielding in the face of the growing boom of wireless communication devices (Wireless) and home devices [39] that emit within the same frequency range of the electromagnetic spectrum, producing noise, interference, and dynamic superposition of signals with a harmful effect on health [40]. The use of nanoreinforced polymers in the protection and attenuation of interference has precedents such as Printed Circuit Board shielding technology [41,42], electromagnetic shielding [43,44], and microwave absorption [45,46] applications. The absorption of electromagnetic waves in nanomaterials occurs by several mechanisms. Wave reflection, absorption, and superposition produce the attenuation of the emitted wave inside the material if this process occurs simultaneously and cooperatively. The strategy used in this work to enhance the reflection capacity and increase the absorption is related to the self-stratification of the GNP.

In this work, we study the electrical, thermoelectrical, and conducted electromagnetic shielding properties of epoxy resin reinforced with GNP, with different specific surface areas. The influence of the sonication times used in the dispersion technique have been analysed, explaining them with morphological changes. The natural GNP stratification, which is usually considered a serious problem, is useful to manufacture interesting materials, whose surfaces shows different electrical and electromagnetic shielding behaviour.

## 2. Materials and Methods

### 2.1. Materials

In this article GNPs type C300, C500, and C750, provided by XGSciences, have been used. The surface area increases proportionally to the decrease in the mean platelet size. The lateral sizes of the different GNPs utilized are 1, 0.5, and 0.3 μm (these data being provided by the manufacturer), while the specific surface area was measured by BET tests in an ASAP 2020 Plus absorption analyser, using N2 at 77K, and the values obtained were 410.93 ± 1.15, 452.63 ± 1.44, and 667.2209 ± 3.42 m^2^/g, respectively. The thickness of the GNPs were analysed by SEM, with the average thickness found to be below 100 nm.

The polymer matrix was an aircraft-grade epoxy resin produced by Huntsman, with a formulation based on Bisphenol A Diglicidyl ether (DGEBA, Araldite LY556), which is a low-viscosity resin with a molecular average weight of less than 700 g/mol and a relative density of 1.17 g/mL, cured with an aromatic amine-based hardener (Araldite XB3473) with a density of 1 g/mL.

### 2.2. Manufacturing of Nanocomposites

**Figure 1:**
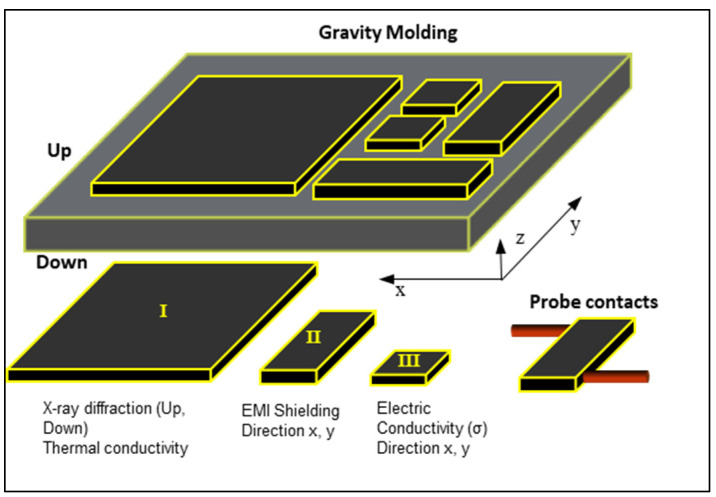
Scheme of the specimens for different experimental test. The identification of the orientation was preserved in each specimen. The red cylinders represent the types of contacts used in EMI.

The GNPs/epoxy nanocomposites were prepared following a process of four general steps: (1) Sonication process of the GNP into epoxy monomer during different times (30, 60, and 120 min) at a frequency of 24 kHz and a power of 400 W with a pulse amplitude of 50%; (2) the hardener monomer is added in the stoichiometric ratio and the mixture is degassed under vacuum with a magnetic stirrer at 80 °C for 15 min to release the gas bubbles trapped in the mixture. GNP content added was 12 wt%, because it is higher than the electrical percolation threshold of this system [47]; (3) the final samples are manufactured by gravity moulding and cured into an oven at 140 °C for 8 h; and finally, (4) the last stage consisted of performing the demoulding and cutting of specimens with the aid of a CNC milling machine. Figure 1 shows the geometry of the different samples machined: Type I specimens are for thermal conductivity measurements and X-ray diffraction test on both sides (Up and Down); Type II specimens are to analyse the propagation of electrical signals; and Type III specimens are used for electrical conductivity characterisation.

### 2.3. Characterization

#### 2.3.1. Electrical Conductivity Test

Electrical conductivity measurements using direct current (DC) were performed by the 4-wire connections method, with the purpose of minimising or eliminating the effects of cable resistance. Type III specimens (Figure 1) were used for this test and comply with ASTM D257. The equipment used was the Source Meter Keithley 2410. The applied voltage was varied between 0 and 500 V, and the recorded current was limited with a compliance of 1 A (up to 25 V) or 0.02 A (from 25 V to 500 V). The automated control system of the Source Meter was performed using LabTracer 2.0 software (Keithley Instruments, Inc.). The electrical conductivity in the *z*-axis, which is much lower due to the GNP self-orientation, was determined using a picoammeter (Keithley 6400), which is capable of measuring currents from 10 fA up to 20 mA. For the calculation of the conductivity, since the I–V relationship is non-linear, the resulting I (V) function was divided into five segments and the linear conductivities obtained were averaged.

#### 2.3.2. Thermal Conductivity Test

Thermal conductivity measurements were performed with the Fox 50 equipment supplied by TA Instrument using Win-Therm 50V3 software (TA Instruments, 159 Lukens Drive, New Castle, DE 19720). The specimens measured were Type I (Figure 1) in the Z direction. The thermal properties of the composite as a function of temperature were conducted with a resolution of 0.1 W/mK.

#### 2.3.3. Joule Effect Test

Joule heating was determined by applying constant direct current (DC) for 1 min at each step to stabilise the temperature until 250 V was reached, using the specimens Type III (Figure 1). The temperature profile of the specimens during the test was recorded using the FLIR E50 thermal camera, programmed to record the ambient, maximum, and average temperature of the specimen. The results presented correspond to the maximum temperature increase, ΔT_max_, obtained with respect to the ambient temperature (20 °C).

#### 2.3.4. X-ray Diffraction

In order to know the degree of self-stratification and exfoliation of GNPs, together with the homogeneity of the GNP dispersion into the epoxy matrix, X-ray diffraction was performed on both sides (top/bottom) on the Type I specimen (Figure 1) with an average thickness of 4 mm. Since the penetration of the radiation is limited, there is no risk of overlapping of the results obtained on both sides. Using ImageJ software for image analysis, X’pert HighScore Plus software to calculate the X-ray attenuation in XRD, and using the images taken by SEM stitching to obtain parameters, it is obtained that the attenuation for C300 is 565 (μm) and for C750 is 607 (μm). So that in both cases the segregation is completely analysed, since their thicknesses are lower, and even greater depths are obtained by performing the calculations assuming the existence of a gradient in the sample, these are included in the analysis. The calculations and results obtained are checked with those obtained by other authors [31,48], confirming their veracity in an approximate way.

The diffractometer used is Panalytical’s X’Pert PRO. The 2Theta scanning program was run from 5 to 90°, in a continuous regime. The copper tube (CuKα, 1.5406 A) was adjusted with the following voltage conditions of 45 kV and a current of 40 mA. The program used in the treatment and identification of the diffractograms was X’Pert High Score Plus (version 3.0, Malvern Panalytical, Enigma Business Park, Grovewood Road, Malvern, WR14 1XZ, UK).

#### 2.3.5. Scanning Electron Microscopy (SEM) and Image Analysis

The samples were cut and embedded in insolating resin for cross-sectional observation. The grinding and polishing procedure for microstructure evaluation was performed according to the recommendations of ASTM E 2015-04. The samples were coated with a thin gold layer of 5 nm, using LEICA EM ACE200 sputtering. The observation of these samples was performed in the Philips XL30 ESEM electron microscope under a high vacuum regime using the BSED (back scattering electron detector). The reduced field of view in electron microscopy does not allow observation of the stratification phenomenon. To solve it, panoramic or stitching images were constructed, obtained from eight consecutive images from the lower edge to the middle zone of the film. The stitching dimensions obtained were 1560 × 400 µm^2^. Digital image analysis was made for determining the agglomerate size and the magnitude of fragmentation or breakage of GNP. The freely available software used for stitching segmentation is FIJI (ImageJ 1.53c, NIH). The samples used to evaluate the GNP exfoliation in the matrix were prepared by cryofracture, using liquid nitrogen as a cooling agent. The notch-directed cross-section fracture was sputter coated with 2 nm thick platinum. The microscope used for this observation was a high-resolution Nova NanoSEM 230, under a high vacuum regime.

#### 2.3.6. Electromagnetic Conducted Signal Absorption Test

**Figure 2:**
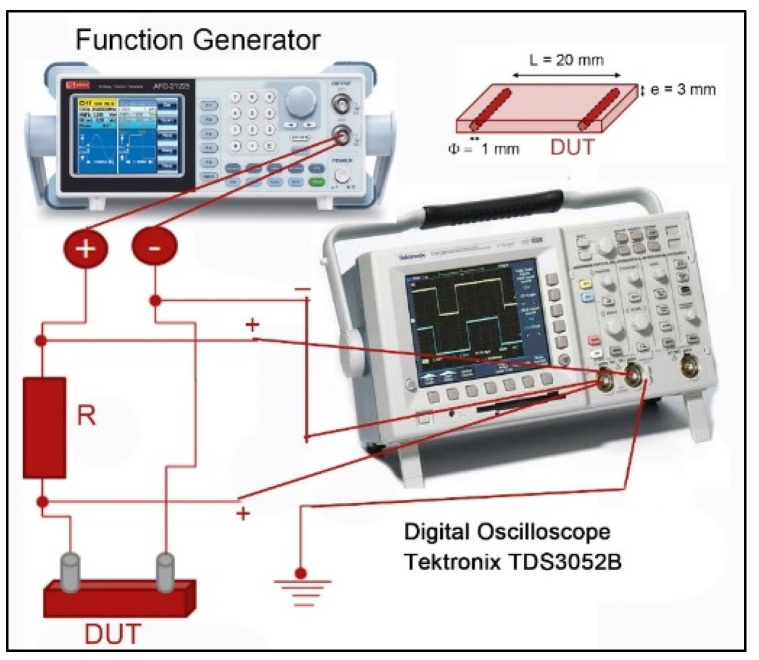
Scheme of the equipment configuration used to study the absorption of electromagnetic waves.

The behaviour of studied nanocomposites against electromagnetic wave transmission has been analysed in a wide range of frequencies (low, medium, and high), in the range from 3 kHz to 30 MHz, in thin materials (<300 μm). Measurements were performed by integrating separate contacts at 20 mm within Type II specimens (Figure 1). The gadget consists of an RSPRO AFG-21225 waveform generator that allows sweeping in a frequency range of 5–20 MHz, an oscilloscope to measure the attenuation of the input signal Tektronic TDS 3052B, and brackets and shielded cables for mounting, as is shown in Figure 2. The devices were calibrated and tested using dissimilar materials to ensure correct measurement [49,50]. In addition, invariant ceramic resistance at working frequencies, whose real resistance value is similar to that of studied materials, has been used to obtain the electrical parameters with the minimum possible impact of noise and maximum sensitivity.

The electrical signal shielding (*EMS*) values are compared considering the real number value (*EMS*), which is determined with the next Equation (1) to calculate the power loss of the signal passing through the material.

(1) $$EMS=20\cdot \log\frac{V_{out}}{V_{in}}$$

Likewise, the amplitude attenuation suffered by the signal (*SAA*) is calculated with the Equation (2), where $V_{in}$ is the input signal amplitude, and $V_{out}$ is the output signal amplitude.

(2) $$SAA=\frac{V_{out}-V_{in}}{V_{in}}\cdot 100$$

## 3. Results and Discussion

### 3.1. Analysis of Self-Stratification in Nanocomposites

**Figure 3:**
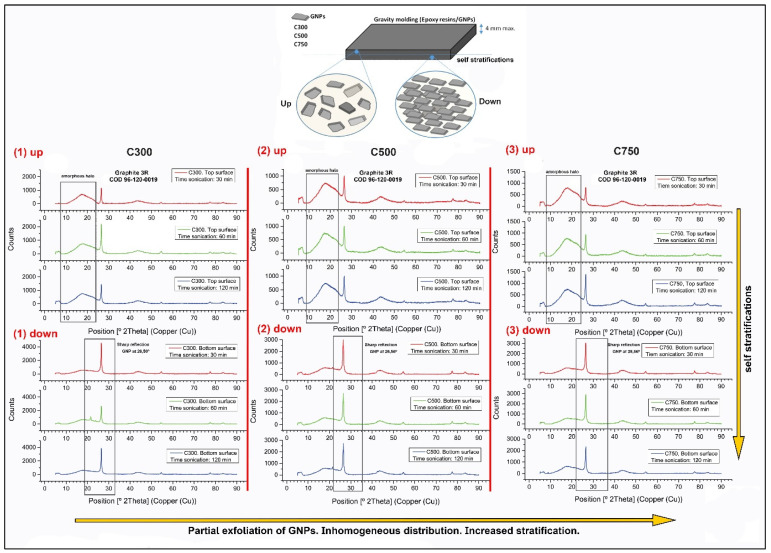
XRD diffraction pattern of GNP/epoxy composites. The spectra obtained are plotted according to the type of graphene used, C300 (1), C500 (2), and C750 (3), according to the surface analysed, that is, up or down, and according to the sonication time of the sample, that is, 30 (red-upper), 60 (green-middle) or 120 min (blue-lower).

The effect of GNP layering or sedimentation as a function of the average graphene area was measured by XRD and is presented in Figure 3, as a function of the sonication time applied. As it can be seen, the first peak belonging to the amorphous halo of the epoxy resin keeps its intensity approximately constant from left to right (upper part), so the type of GNP does not affect the formation of the GNP-poor upper layer. However, the analysis at the top also shows that the GNP content decreases when moving from left to right, indicating that reducing the average lateral size, and increasing the surface area of the graphene produces greater sedimentation, reducing the graphene content on the upper surface. Self-stratification is also verified by the difference in intensity of the main peak between the upper and lower surface belonging to graphite at 26.5°, which corresponds to the (0 0 2) plane COD 96-120-0019 [51,52]. The intensity of this peak on the lower surface is twice as high with respect to that recorded on the upper surface. Moreover, the presence and coincidence in intensity of peaks 2θ = 54.6°, 77.4°, and 83.5° belonging respectively to the (0 0 4), (1 1 0), and (1 1 2) planes in all spectra, demonstrate that exfoliation is residual, unlikely, and exists to an insignificant extend.

The non-uniform distribution of the graphene in the thickness of the sample negatively affects the mechanical properties, since in the bottom zone a large number of agglomerates are generated, inducing a situation similar to the one that would be caused by a bad dispersion [53]. However, it can generate interesting applications or advantages by also modifying the electrical and thermoelectric properties; for example, generating preferential paths in the conductivity for certain applications in multifunctional systems, such as localised heating, is useful. In addition, the selective exploitation of thermoelectric properties near the bottom surface improves the thermal transfer to the substrate, while slightly heating the top surface reduces dilatometric changes and improves compatibility with other coatings of lower thermal resistance.

**Figure 4:**
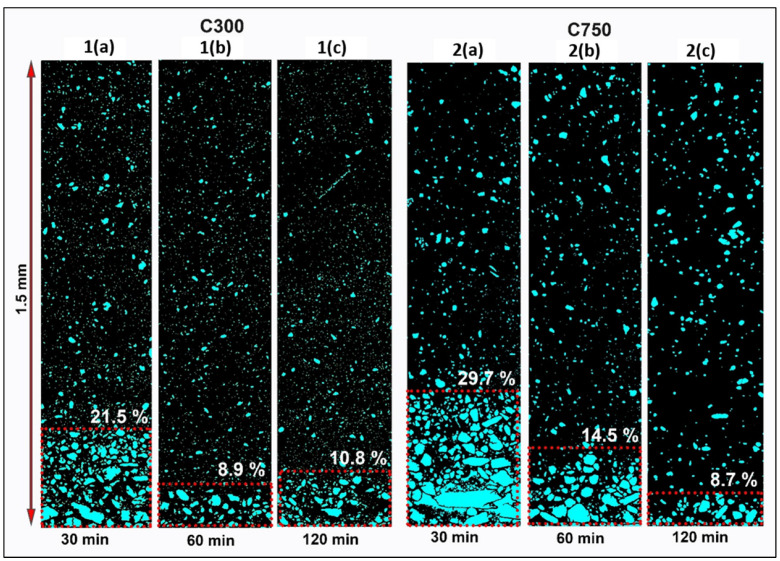
Coloured panoramic SEM (Stitching) images show the percentages of self-stratified GNP C300 (1) and C750 (2), applying different times of sonication 30 (a), 60 (b), and 120 min (c). The percentages indicate the height of the sedimentation layer.

The dispersion state of GNPs was evaluated by SEM, using previously prepared specimens in their cross section. Figure 4 shows the panoramic images (stitching) belonging to two systems based on C300 and C750 (only the extremes are analysed), dispersed using sonication times of 30, 60, and 120 min. Panoramic imaging of the areas near the bottom surface of all specimens was performed using the backscattered electron detector (BSED). The contrast by atomic weight difference produced by the detector allows differentiation of the layering and sedimentation of the GNP agglomerates near the bottom surface. Segmentation of the images has allowed us to quantify the dispersion in a region of 1.5 mm from the bottom edge.

As expected, the self-stratification and agglomerate formation are more intense in the C750, as indicated by Stokes’ law. The agglomerates formed in the bottom of this sample are twice the size of those observed in the C300 sample due to their higher specific area. Agglomerate formation and self-stratification are reduced as the sonication time increases, because of fragmentation/breakage of the graphene nanoplatelet agglomerates. On the other hand, this method seems not to produce exfoliation, if not fragmentation of the GNP, so it does not improve the connectivity between graphene platelets. Consequently, it deteriorates the electrical and thermal conductivity of the composite material, as will be shown in the next section.

**Figure 5:**
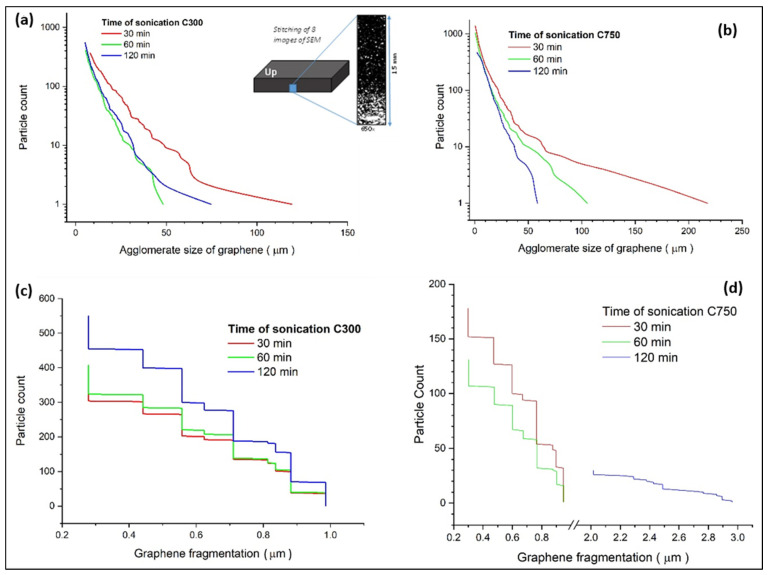
Analysis of agglomerate size and fragmentation state for different dispersion times and graphene types (C300 and C750). Agglomerate size evolution with sonication time for GNP C300 (**a**), and for GNP C750 (**b**), scheme of analysis location, and GNP size evolution for GNP C300 (**c**), and for GNP C750 (**d**).

The segmentation and analysis of the dispersion of nanocomposites have allowed us to quantitatively evaluate the state of agglomerates and GNP fragmentation during processing (Figure 5). The data obtained were filtered considering that particles greater than 1 μm were designated as agglomerates, while smaller than 1 μm would be considered fragments of the nanoplatelets of the initial graphene. This separation allowed excluding the agglomerates formed during processing, verifying that the longer the sonication time, the more intense the fragmentation and the lower the connectivity.

The effect of sonication is different for each type of graphene, observing that for C300 the fragmentation of the agglomerates mainly occurs in the first 30 min (Figure 5a), while in C750 the fragmentation of agglomerates occurs during the 120 min test (Figure 5b). It is also important to note that between 60 and 120 min the C300 system undergoes an increase in the size and number of agglomerates, which allows us to affirm that sonication is ineffective, probably due to an increase in density. The energy provided during sonication is used by the system to facilitate Brownian motions that increase the probability of intricacy between the GNPs, agglomerating them.

On the other hand, it is confirmed that the lateral size after sonication of C300 GNPs is larger than C750 (Figure 5c,d). Moreover, it is observed how for C300 (Figure 5c), an increase of the sonication time from 60 to 120 min leads to a higher fragmentation of the GNPs, so that the sonication energy is able to break the GNPs, which does not occur for 30 and 60 min because they almost all form agglomerates in the sedimentation layer. Finally, from Figure 5d it is concluded that an increase in sonication time generates GNP sizes smaller than 0.3 μm, which are not shown because this is the minimum dimension that the SEM used can study. This implies that an increase in the sonication time generates a breakage of the GNPs, decreasing their lateral size.

**Figure 6:**
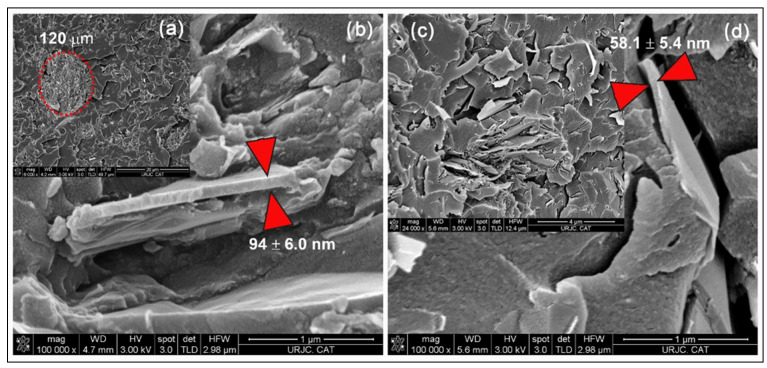
FEG-SEM images of GNP/epoxy composite reinforced with C300 (**a**,**b**) and C750 (**c**,**d**).

The analysis of the cross section by Field Emission Gun—Scanning Electron Microscope (FEG-SEM) applying oriented cryofracture allowed the evaluation of the dispersion of GNPs in the matrix and, at high magnifications, to verify the state of graphene exfoliation. Figure 6 shows the images obtained after carrying out a progressive sequence from the agglomerate in the self-stratified zone to the detail of the cross section of non-exfoliated graphene packing. It is observed that the non-exfoliated graphene platelets present thicknesses greater than 50 nm in cross section. This could generate how the conductive properties, both thermal and electrical, are lower than expected, being overcome with the use of other types of graphene [31], as will be confirmed in the next section. In addition to the thickness, the fact that the graphene in this case is not functionalised also plays a role, which results in a weak interface.

### 3.2. Electrical and Thermal Conductivity Analysis

**Figure 7:**
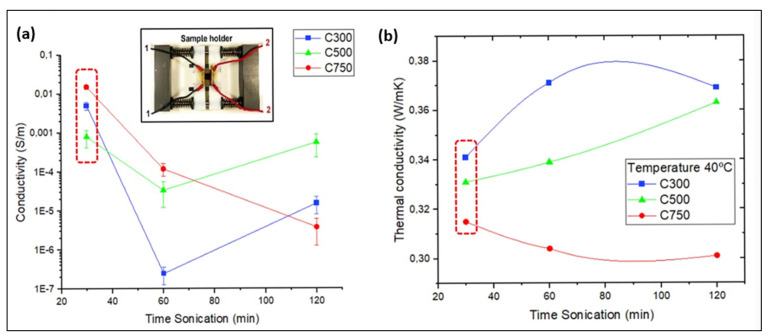
Electrical (**a**) and thermal (**b**) conductivity of GNP/epoxy composites, applying different ultrasonic dispersion times 30, 60, and 120 min. The red box indicates the conductivity values for the optimum sonication time for which the best thermoelectric properties are obtained. The image of graph (**a**) shows the conductivity measuring instrument at four points.

Figure 7 shows the electrical and thermal conductivity of the composites depending on processing sonication time and the type of graphene. It is important to highlight that the electrical conductivity is studied in the direction of the plane, while the thermal conductivity is studied in the z direction, to know its potential application in heated coatings. The results reveal that, when using C300 and C500, an increase in the sonication time produces a drop in conductivity, and this drop is determinant between 30 and 60 min. This drop in conductivity can be justified by the amorphization transformation undergone in the graphene structure [47]. However, between 60 and 120 min, a rise in the electrical conductivity is shown, which may be due to the stabilisation of the appearance of defects and amorphization while continuing to generate a better dispersion [54]. This last hypothesis is justified by the results of XRD, decantation size, and fragmentation observed for C300 in the stratification analysis section (Figure 3, Figure 4 and Figure 5), since it is observed therein that with increasing sonication time from 60 to 120 min, the decantation size and amount of GNPs at the bottom grows; there is not agglomerate breakage, but fragmentation of GNPs is relevant.

It is interesting to note that C300 presents a greater slope of conductivity drop compared to C500, which means that amorphization and GNP fragmentation occurs more easily, which could mean that electrical stabilisation is reached earlier. This greater ease of amorphization would be justified by a lower presence of impurities, while fragmentation is more effective as it has a lower tendency to form agglomerates (due to its smaller specific area).

The results obtained when C750 is used show that the conductivity drop is visible between 30 and 120 min, losing slope as time goes by due to the decrease of the lattice damage produced on the structure. It is interesting to note that these values, together with those observed for C300 and C500, allow us to make a strong hypothesis about the different influence of the surface area and lateral size on the damage stabilisation time, since as the surface area increases and the lateral size of the GNP type decreases, the time until conductivity stabilisation increases. This behaviour observed for C750 is justified by the results observed in XRD (Figure 3), SEM stitching (Figure 4), and fragmentation analysis (Figure 5). In both changes of times, from 30 to 60 min, and 60 to 120 min, the size of sedimentation and the quantity of GNPs are lower; additionally, the fragmentation is high, although it is lower between 60 and 120 min due the increase in viscosity.

A greater specific area, maintaining constant dimensions, seems to lead to a probabilistic increase in the existence of greater rupture events, which from the mechanical point of view are favoured by the possibility of the appearance of greater net stresses linked to larger dimensions, such as bending moments. Increasing the dimensions, for a constant geometry, would lead to the same. However, when observing the induced damage as a linear combination of lateral size and specific surface area, the slope accompanying the lateral size must be greater for the experimentally observed data to have justification, since if both slopes were equal, the one that would take longer to present an increase in conductivity due to exfoliation would be C500. In contrast, if a greater weight is given to the lateral size of the GNPs, the electrical behaviour with the increment of sonication time has justification.

The above is feasible from the physical process point of view, since the collision of a bubble during sonication, which is what gives rise to the external forces acting on the GNPs, has an area of action around few microns (0.1–25 μm) as observed in papers [55,56,57] analysing the evolution of bubbles generated during the sonication process under conditions similar to those present in the current project. Moreover, considering how the dissimilarities between the resin loaded with GNPs and the fluids used in the articles [55,56,57,58] affect the process, it can be justified that the smaller size is possible because our fluid presents a much higher viscosity, and lower specific heat (and therefore specific heat ratio) and density. The fact that the zone of action of the bubble jet is in the order of microns means that as the lateral size of the GNPs decrease, there is a lower probability of fragmentation as the generation of a bending moment is less probable. This explains the difference in behaviour between the types of GNPs together with the rest of the justifications.

To the above, it must be added that the manufacturer provides the elemental concentration percentages of the different GNP types, indicating that the highest presence of impurities is in C750. This implies that initially the effect of amorphization and damage in its lattice is less because there are particles that are not GNPs present, that means that over a longer period of time you can cause damage. Ultimately, C750 structure is the most suitable to lengthen the effect produced by the damage of sonication.

In summary, Figure 7a shows that the highest conductivity values occur for specimens with C750 with sonication times of 30 min, followed by C300, and finally C500. The most conductive specimens have in common a minimum sonication time, which allows affirmation that sonication is not adequate beyond 30 min. It can even be doubted, because the conductivity values for times shorter than 30 min are unknown, whether it is really beneficial to perform such a long sonication stage when there is self-stratification. In addition, the longer sonication times end up generating an inversion of the conductivity domain according to the types of GNP, converting the one that at short times was the least conductive into the most conductive, and vice versa. For times of 60 min there seems to be an intermediate transition phase in which C500 already presents a higher conductivity than C300, so that C500 seems to present a great resistance to the drop in conductivity with the passage of time compared to the other types of GNP.

The highest conductivity obtained for C750 is higher for 30 and 60 min, because during the first 30 min, this type of graphene received less damage and amorphization, which has been already justified. Moreover, its effective conductive volume linked to the sedimentation size is higher than in C300 and C500 because the bigger specific area results in high agglomeration (bigger size of agglomerates results in higher sedimentation velocity, according to Stokes’ law). However, sonication times above 60 min generate an increment of sedimentation size in C300 and C500, which produces as a consequence a higher conductivity by its relation with the effective conductive volume. Bonded to this, C750 continues losing conductivity, its value falling under the others due to the reduction of conductive volume (the electrical resistance augment) and increase in fragmentation.

A different behaviour was observed in the thermal conductivity measurements in the Z direction (Figure 7b). The measurement is made from side to side of the specimen in the Z direction, so that the sedimentation is very influential in the result. Knowing the thermal behaviour in this direction, it is interesting to check the potential use of the material as a heating coating.

In the C300 and C500 systems, the decantation degree decreases with the sonication time, resulting in a greater amount of GNPs in the rest of the sample, which generates a local increase in the thermal conductivities. Added to the previous, it is known that there is an increase in viscosity as the sonication time increases [33], which generates a better relationship between the exfoliation induced by the cavitation forces and the breakage of the GNP, supporting this increase in thermal conductivity. However, in the C750 system it is the opposite, since increasing the sonication time leads to a decrease in thermal conductivity. For this type of graphene, although it is observed that there is a progressive elimination of the self-stratification zone with the sonication time (Figure 4), it is observed that the GNP is not properly dispersed throughout the thickness, finding agglomerates of larger sizes than those observed for C300, which are becoming more and more uncommunicated with the pass of sonication time (Figure 4b). This, together with the increment of GNP fragmentation that decreases the lateral size, are the main causes of the thermal conductivity decrease with increasing sonication time [59].

It can also be concluded that the thermal conductivity decreases with increasing surface area and decreasing lateral size, that is, when we go from C300, to C500 and C750, since a higher specific surface area can lead to a higher porosity of the GNP, a defect that impairs the thermal conductivity [47]. Moreover, the smaller lateral size of nanoplatelets negatively influences the thermal conductivity value [60], not only because it decreases the intrinsic conductivity of GNPs, but also because it induces a lower increase in the glass transition temperature of the compound [61]. The results obtained from X-ray diffraction (Figure 3) also explain that the thermal conductivity in C750 should be the lowest, since lower amounts of GNPs are observed in the upper zone. This generates that the zone with the lowest amount of GNPs (the upper one), which act as the limiting part of the system, act as an insulating thermal barrier.

As a conclusion, it can be stated that the composites nanoreinforced with GNPs improve their thermal conductivity for longer dispersion times due to the decrease in the size of the decantation zone, except for the composite type C750, because it has a heterogeneous distribution of graphene in the Z direction (Figure 1). It is also confirmed that an increase in the specific surface area and a decrease in the lateral size imply a lower thermal conductivity.

**Figure 8:**
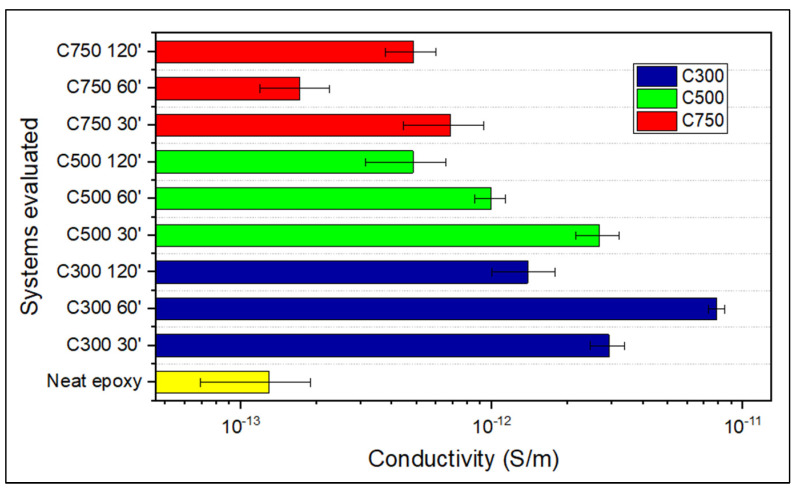
Electrical conductivity in the Z-direction of GNP/epoxy composites.

The results obtained from the picoammeter are shown in Figure 8, which shows that all the samples have extremely low values of electrical conductivity in the Z-direction with respect to the conductivities measured on the XY-plane, although in all cases, as it was expected, the conductivity of the neat epoxy resin was improved.

It can be concluded that increasing the sonication time from 30 to 120 min, for all types of GNPs, generally results in a drop in conductivity, which is related to the amorphization, and the decrease in lateral size (fragmentation) generated on the GNPs during sonication. These two parameters are more influential than the dispersion obtained by the agglomeration fragmentation and consequent decrease in thickness of the segregated zones. This is because the XRD spectra indicated that this greater dispersion did not prevent the existence of the upper dielectric layer of low GNP content, whose GNP content is considered as a limiting parameter on the electrical conductivity in the Z direction.

The fact that the conductivity for equal times is higher in C300 than in C500, and superior in C500 than in C750, is justified by stating that the larger lateral size of C300 and C500 causes the GNPs to have a greater difficulty to decant. Because a larger size implies a greater hydrodynamic resistance, this translates into a greater amount of GNPs in the upper zone of the sample. This higher quantity of GNPs in the upper zone is confirmed with the XRD analysis (Figure 3).

### 3.3. Joule Heating

The results of the temperatures reached when different voltages are applied in all the specimens and in both directions (x,y) contained in the plane have been obtained; however, only the results of the specimens that have given off noticeable heating will be shown. These samples are those manufactured with 30 min of sonication, because their higher electrical conductivity implies a greater Joule effect due to the increase of the current flow [62]. It is important to note that the powers used for heating do not exceed 10 W at any time, so the system shows good energy efficiency.

To analyse the thermal behaviour of the fabricated systems, the activation voltages have been studied (Table 1), which represent the potential difference at which two different zones of the material have to be imposed to mobilise the charges sufficiently to generate the Joule effect. This parameter is related to the ease of each system to be heated, that is to say, a low activation voltage indicates greater ease of heating of the system. In this case, the activation voltage has been defined as the voltage necessary to observe in the thermal chamber an onset of heating of the sample above 10 °C, which is the minimum temperature required in some applications of industrial interest [63,64]. It is important to highlight that the activation voltages have been defined as intervals which collect all the values of activation voltages obtained, since several specimens have been analysed in both directions belonging to the plane (x and y).

All the above implies that only systems with a sonication time of 30 min are of interest for their application in industry, since longer sonication times imply too high values of necessary voltage, so only the systems that have been manufactured with that sonication time will be analysed.

**Figure 9:**
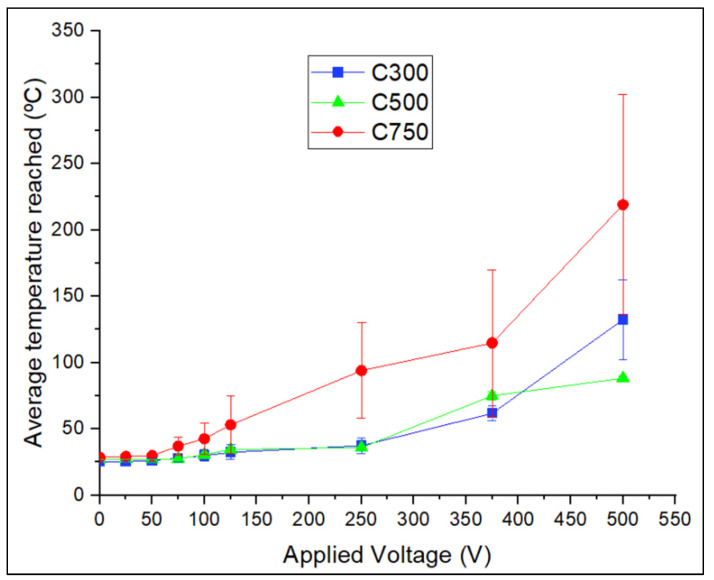
Average temperature reached as a function of the applied voltage for GNP/epoxy composites, applying 30 min of sonication.

Figure 9 shows that the system with C750 has the highest Joule effect heating capacity, followed by C300. This a priori makes physical sense with the results obtained in the characterisation of electrical conductivity in DC, since a higher electrical conductivity leads to a higher current flow (intensity), and it is known that heat generation grows exponentially with increasing current intensity [62].

The specimens incorporating C750 show a greater Joule effect because the associated impurities cause a lower thermal conductivity [37,65], which locally increases the heat, resulting in a greater intervention of phonons in a localised area. This phonon intervention would explain the early onset of the exponential trend since the electrical conductivity is linked to the multiphonon-assisted Joule effect [66]. The fact that the C750 system shows a higher error in the values obtained is due to the existence of a larger number of specimens that presented preferential percolated conductive paths. Apart from the above, it is known the heat actually accumulates to a greater extent in C750 than in C300 due to lower thermal conductivity (Figure 7b). That, together with the higher electrical conductivity of C750, make up the basis for the difference between the temperatures reached. The existence of an initial trend with a greater slope is justified by the difficult heat transfer to the outside.

**Figure 10:**
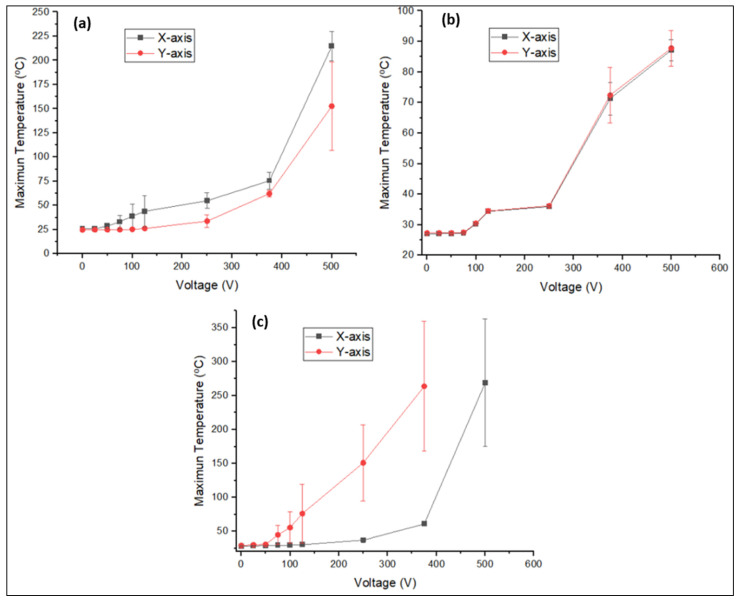
Heating results of the GNP/epoxy composites according to the type of graphene used and direction of bias imposition. Joule effect graph is plotted for the C300 30 min system (**a**), the C500 30 min system (**b**), and for the C750 30 min system (**c**).

All the analysed specimens have been plotted according to the type of GNP, distinguishing the axis on which the potential difference was established (x or y), and for a constant sonication time of 30 min (Figure 10), since, as previously mentioned, this is the time that has allowed for reaching the highest temperatures.

Figure 10a,b shows that the Joule effect in the specimens containing C300 and C500 depends weakly on the axis on which the bias is established. This dependency is observable on the difference between the maximum temperatures reached, highlighting that this difference is not high if the deviations of the different specimens analysed are taken into account. The above results show that the processing conditions of C300 expose that there is a slight anisotropy of the thermoelectric properties within the plane. This would not be a problem if you want to do Structural Health Monitoring (SHM) using the Joule effect, but it would have to be taken into consideration when doing SHM. It is important to note that in all the graphs in Figure 10, the wide errors are associated with the existence of preferential conductive paths, due to how their formation is uncontrolled.

Using C750 (Figure 10c) allows for reaching temperatures twice those obtained by C300, keeping the bias and activation voltage values similar. Here the difference is that there is one single zone instead of two in which the presence of preferential conductive paths has been detected. Therefore, the homogeneity previously observed for C500 is broken when using C750, since each specimen shows a unique heating, i.e., the system shows unpredictable behaviour from the modelling point of view. The origin of the formation of the preferential conductive paths may be linked to a process of physical self-organisation due to the appearance of preferential zones for the occurrence of electrostatic interactions that generate agglomerates.

**Figure 11:**
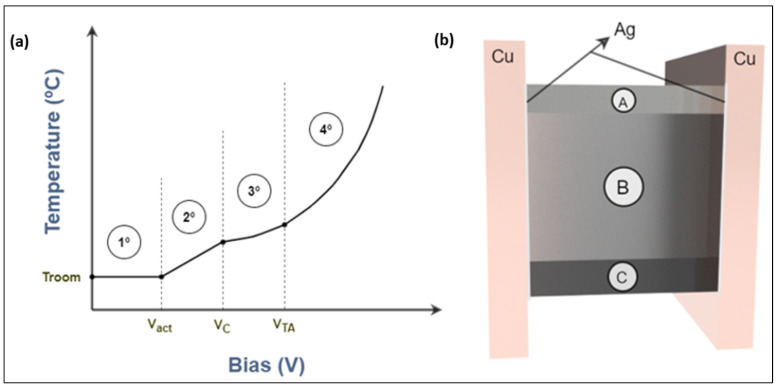
General temperature tendency graph with the different stages (**a**) and scheme of nanomaterial with different sedimentation zones (**b**). It is possible to observe the three characteristic voltages that represent the change of trend ($V_{act},V_{C},V_{TA}$).

In all cases, the existence of four stages has been observed (Figure 11a). First, there is a voltage range for which the temperature does not vary, and second, where the temperature increases linearly. Then, a potential tendency for an increasing bias (whose exponential value depends on the type of GNPs used) is observable. Finally, a fourth, in which the slope of the potential function increases progressively and is corrected by means of an exponential function depending on the temperature of the material itself (as a product of functions).

Before justifying the existence of these four stages, it is hypothesised that there are only two paths for the transport of charge carriers within these materials (zone “A” is considered to have insufficient conductivity), which could be visualised as two parallel paths (Figure 11b). The first would be the discontinuous paths (or capacitive path), which are generated in the areas (Figure 11b zone B) where the material is above and near the percolation threshold. In this type of pathway, we have tunnelling electron transport channels through the epoxy separating the GNPs in the nanocondensers of the system, which dominate for a bias value below a voltage, which we will call critical voltage. Secondly, we have the additional pathways to the previous one formed by the cold emission effect, hopping conduction, and thermionic emission that arise when the critical voltage is exceeded. The next transport path is the so-called continuous path (or resistive path), which appears in areas **(**Figure 11b zone C**)** of the material above the percolation threshold, where all the above named types of conduction occur with possible ohmic drift conduction in the preferential conductive paths [67,68]. All these conduction methods depend on the differently applied bias voltage (Table 2). Area “A” of Figure 11b represents the zone that has not attained percolation threshold.

It is important to note that temperature plays an important role (Table 2), since the increase in temperature leads to an increase in the phonon population. This causes the appearance of hopping conduction (since it is a process that requires thermal activation in which electrons exchange energy with the phonon system), leading to an increase in conductivity (negative temperature coefficient). It also allows the occurrence of thermionic emission and affects the cold field emission [71]. However, in continuous paths (and in conduction through the GNPs or their agglomerates) the effect is directly proportional to how the temperature modifies the GNP conductivity, and in this case, the negative temperature coefficient of the resistor is depreciable compared to that in discontinuous paths [73].

In the first stage, the material shows very low conductivity, so no energy dissipation is generated, and the temperature does not vary. The resistance of the continuous (resistive) path is large, and for the given voltage, the current is of the order of microamperes, so the Joule effect is not generated or is negligible. The tunnelling effect condition is hindered by the Coulombic repulsion energy generated in the nanocondensers of the discontinuous (capacitive) paths [74]. In this first stage, drift (shallow) and tunnel effect conduction (when V < 50 V) are associated with a linear I–V relationship (Figure 11a, A to B), although for some cases it has been observed that tunnel effect conduction can be associated with a third order polynomial relationship for V > 10 V [69]. The observed results indicate that conduction below the activation voltage ($V_{act}$), approximately 50 V, does not induce heating.

In the second stage, linear temperature growth is achieved after exceeding the activation voltage. This linear conduction (Figure 11a, B to C) arises because there is ohmic conduction by drift in the continuous or resistive vias. To this is added the linear behaviour of tunnel transport predicted by the mixed NLRRN + DRRN model at voltages below the critical voltage [75,76], which occurs in the conductive or capacitive vias. The tunnel effect can be taken as linear, since in this case up to about 100–125V no potential I–V trend is observed. The measured temperature growth should have second order potential due to the linear I–V relationships (Table 2); however, with the appropriate coefficients a potential trend can be approximated to a linear one.

In the third stage, the potential growth is available. The critical voltage ($V_{C}$) has been exceeded and, according to the NLRRN (nonlinear random resistor network)–DRRN (dynamic random resistor network) mix model, additional conductive paths formed by the cold emission effect are activated (Figure 11a, C to D). This effect occurs due to the strong electrostatic fields generated between the GNPs (nanocondensers) in the discontinuous path. In addition, hopping and thermionic emission conduction can be taken into account, although both are exponentially temperature dependent, being depreciable for values below the thermal activation voltage ($V_{TA}$). Tunnelling conduction above the critical voltage, i.e., at this stage and the following one, has an associated quadratic–exponential I–V relationship (Table 2), which explains, together with the quadratic function of the field cold emission, the polynomial trend of the T–V relationship (Figure 10a). The temperature can generate a variation of the temperature obtained because the field emission has a second order potential I–T relationship.

In the final stage, a potential–exponential growth is observed (Figure 11a, D to E). The increase in the slope of the potential trend is due to the fact that the increase in temperature allows a hopping conduction (deep conduction) as the phonon population increases, and to a lesser extent a thermionic emission [68]. Interaction with phonons in the insulating matrix induces current growth [74], basically phonon-assisted electron hopping is observed, where phonons facilitate electrons having energy higher than the activation energy gap of the nanocomposite [66]. Other authors say that this behaviour is a consequence of the polymer chains and impurity ions acting as traps for charge carriers to move through the hopping process [77].

**Figure 12:**
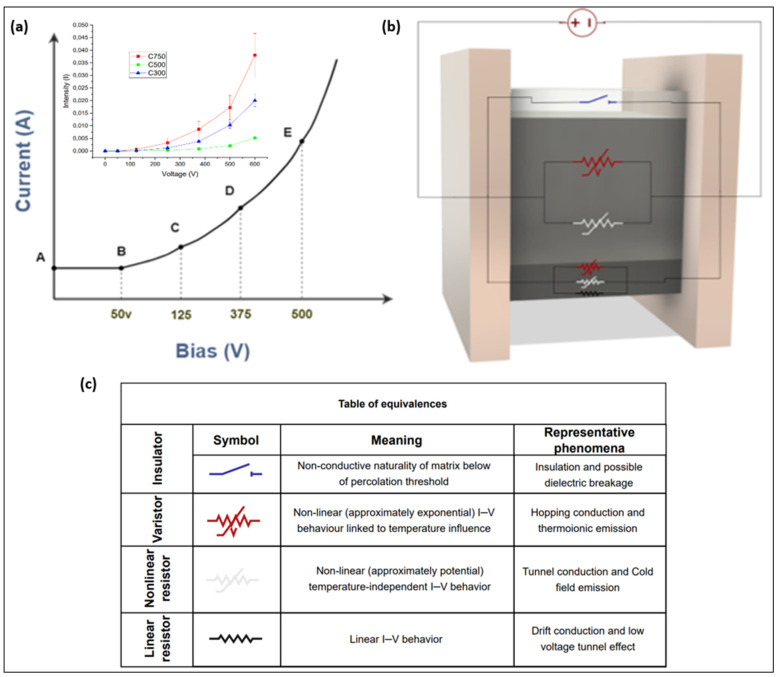
General I–V tendency graph (**a**), general equivalent circuit scheme (**b**), and table of equivalences for the equivalent electric elements representative conductive phenomena (**c**).

Figure 12b shows the general equivalent electrical circuit proposed for the behaviour observed in the polymer nanocomposite with sedimentation. Each zone is compounded by a basic circuit, where each component represents different effects, and the meaning and phenomena are linked to the components described in the table in Figure 12c. Components are situated as parallels because the phenomena can occur at the same time, and each phenomenon can create new conductive paths for electrons.

The existence of a possible lower overlapping of the GNPs, which results in less viable area for the generation of the tunnelling effect, explains why there are different critical voltages for specimens of the same system, i.e., the critical voltage could depend on how the GNPs are oriented with respect to each other, as occurred for the electrical resistance in the conductivity model proposed by Sánchez-Romate and co-workers [31]. The activation and critical voltage would depend only on the resistance of path, which could be found mainly in sedimentation zone. Finally, it is important to note that the tunnel effect is mainly generated in zone C (Figure 11b), where the tunnel resistivity is lower due to the low tunnel distances between GNPs. This explains why in the thermal chamber a faster heating is observed in the sedimentation zone.

**Figure 13:**
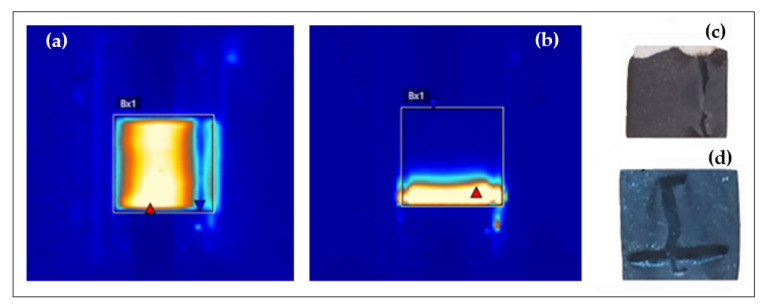
Thermo-photograph images of a sample without the presence of conductive paths and uniform heating (**a**), and with conductive paths and isolated heating (**b**). Photographs of the preferential conductivity paths in specimens C300 (**c**) and C750 (**d**).

**Figure 14:**
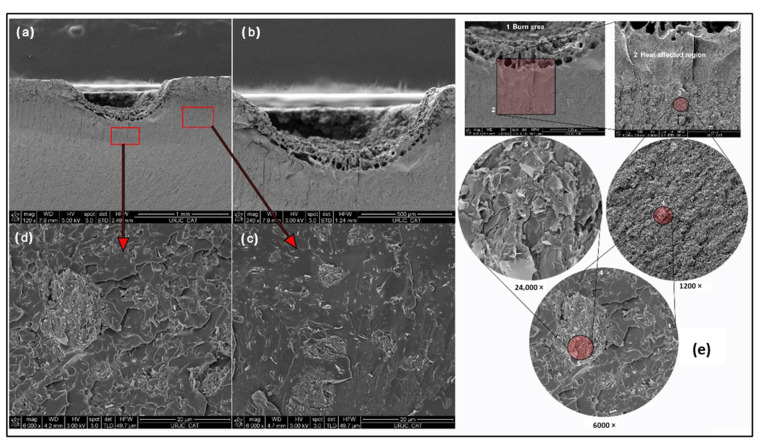
FEG-SEM Images of GNP/epoxy composites reinforced with C750. (**a**) Location of the crater generated by combustion in the preferential conductive path, (**b**) close-up of the crater, (**c**) GNP in the zone outside the combustion crater, (**d**) GNP in the zone where the preferential conductive path exists, and (**e**) detail of the crater zone at different magnifications (1200, 6000, and 24,000×). Red arrow is used to indicate the area that has been magnified.

In the videos collected by the thermal camera it is verified that the start of heating is sometimes not generated in the whole area as is common (Figure 13a), observing this in a marked line (Figure 13b), so the system achieves a faster heating. This is due to the fact that heat cannot be transmitted at a similar speed as the rest of the specimens due to the lack of graphene in the areas near the preferential path (Figure 14). This generates a faster increase in temperature, which in turn raises the participation of phonons in the electrical conduction by momentum transmission to the electrons [78]. Thus, a feedback loop is generated, in which the excess of graphene concentrates the charge, the lack of graphene around it concentrates the heat, the conductivity increases due to the increase in heat, and the heat increases due to the growth of the conductivity.

It is predicted that the origin of this phenomenon is found in the difference in the GNP content between the preferential conductive path and other areas. The low graphene content in the external areas causes thermal isolation due to the lower thermal conductivity coefficient induced. Moreover, the high graphene content generates a lower distance between GNPs, which is translated into high tunnel effect conductivity (high current flow in this path). These two phenomena generate the feedback loop previously mentioned, and as an effect of this appears before the thermal activation condition, in this case hopping conduction, it justifies the existence of lower critical voltage in this path.

For better understanding the origin, this phenomenon was studied using FEG-SEM microscopy (Figure 14). Observing that the conductivity around the path is limited by the lower GNP content found (Figure 14a–d), the hypothesis given above was correct. That is, the heating line pattern is caused by a path where the percolation threshold has been exceeded, while in the rest of the sample in the same layer, the GNP content is lower and therefore exerts a greater opposition to the passage of current.

Figure 14e presents the images of the microstructure of the cross section of one of the tested specimens belonging to C750. In this progressive sequence of images taken at different magnifications, it is possible to observe: the carbonized region, the area affected by the heating, the state of the graphene dispersion in the areas surrounding the integrated conductor, the agglomerates present in the layered area, and the inner part of the agglomerate. In summary, although the 30 min sonication cycle leaves a large number of agglomerates, the platelets present a homogeneous distribution in the space between the agglomerates.

**Figure 15:**
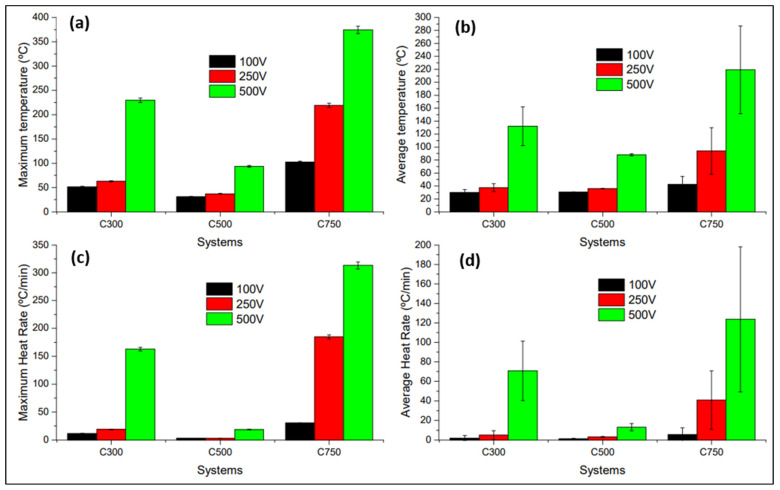
Values of maximum temperature (**a**), average maximum temperature (**b**), maximum heating rate (**c**), and average maximum heating rate (**d**) achieved by the application of different voltages (100, 250, and 500 V) on different systems manufactured for 30 min of sonication.

Finally, in order to compare the Joule effect generated by each type of GNP, Figure 15 shows the values of maximum temperature and heating rate reached by the systems analysed, as well as the averages of the two previous values.

The results shown in Figure 15a allow us to conclude that the maximum temperature reached for all the voltages of interest is linked to the use of C750, reaching up 374.4 °C at the maximum voltage. Additionally, those in Figure 15b indicate that the average maximum temperature is also obtained using C750 for all the voltages evaluated, at 219.1 °C. The graphene that records the worst temperature values is C500, while C300 exhibits intermediate values between the two previous ones. Figure 15c shows that the maximum heating rate value is achieved using C750 for all voltages, with a value of 313.3 °C/min for 500 V. On the other hand, Figure 15d shows that when the heating rates are averaged, C750 is still the optimum system, but with a lower maximum of 104.3 °C/min. Again, in this case it is C500 that presents the worst values, and C300 is in between the other GNP types.

### 3.4. Electromagnetic Signal Absorption Behaviour in Nanomaterials with Different Specific Surface Areas

The values of conducted electromagnetic shielding at low, medium, and high frequency, that is, between 100 Hz and 20 MHz, have been obtained for all type I specimens, which allows us to have information on how the attenuation of the electromagnetic signal propagated by conduction varies in different material directions, and therefore whether the transmission of the same is independent of the heterogeneities that the material may present, such as the different segregation thicknesses obtained in the different systems.

**Figure 16:**
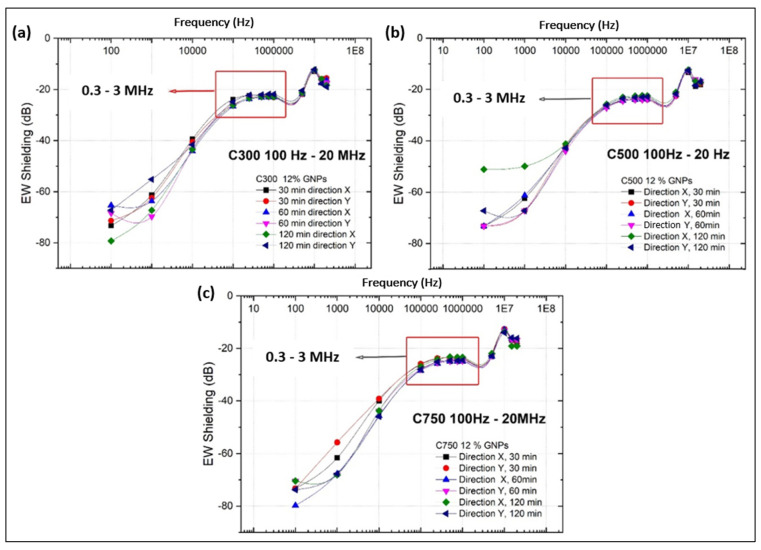
Electrical signal shielding value according to sonication time and transverse propagation direction in the low, medium, and high frequency bands for GNP C300 (**a**), C500 (**b**), and C750 (**c**).

Figure 16 shows how in C300 there is different behaviour between the signal propagation in the X and Y directions. This difference increases as the sonication time is increased, but for all times it is observed that the shielding difference between the different directions decreases with increasing frequency until reaching a zone where the shielding is independent of direction, sonication time, and frequency. This zone is represented by the mid-frequency band 0.3–3 MHz.

In C500 the same behaviour is also observed of the shielding between the signal propagation according to the direction increasing with the increment of sonication time. Again, the shielding difference decreases until reaching the stability band of 0.3–3 MHz. For C750 it is not observed that the shielding variation between different directions depends on the applied sonication time, but again the existence of the stable shielding zone in the mid-frequency band is observed.

It can therefore be concluded that there is a medium frequency band between 0.3–3 MHz in which the shielding value is independent of the sonication time, the direction of signal propagation, and the frequency, since there are variations of the shielding value of less than 1%. If the variations of the output amplitude versus the signal input amplitude are also analysed, statistically, values lower than 0.5% are obtained. This enables potential SHM applications in this frequency range, since the variations of the deformation state can be related to the variations of the shielding or amplitude ratio in this mid-frequency band. This variation of conductive EM shielding with the deformation has been verified by performing a three-point bending test, concluding that the sensitivity is low due to the high rigidity of the epoxy matrix (it is only possible to observe a significant difference in the signal when the material suffers damage, such as cracks).

The behaviour shown in Figure 16 reveals three stages, which coincide with the intervals of low, medium, and high frequency. For low frequency, the drop in conductive shielding with increasing frequency is directly related to an increase in the conductivity of the nanocomposite. The conductivity of the nanocomposite increases with increasing frequency because the large amount of GNPs in the matrix produces a strong Maxwell–Wagner–Sillars effect, which disappears as the charges accumulated at the interfaces decrease, resulting in a drop of dielectric constant (Ɛ’), linked to an increase of dielectric loss (proportional to the electrical conductivity) [79]. This means that the dipoles inside the microcapacitors disappear, giving a high mobility of the carriers through the hopping conduction mechanism by using the defects of the polymeric chains [80]. This conduction is possible because the losses produced by the impossibility of the dipoles to adapt to the E field provide energy to the medium, increasing the phonons [81].

In the mid-frequency case, a frequency-independent shielding is observed. This is due to the fact that the MWS effect no longer occurs at these frequencies, thus having frequency-invariant electromagnetic properties. For the high frequency range, there is a minimum in the shielding, where the drop and subsequent rise of the conductive shielding indicate that it is due to a passive disturbance of resonant nature.

In addition, in order to study the influence of the sonication time on the shielding value versus frequency, the type of GNP is kept constant, an average of the values in the different directions is calculated to eliminate the variability of the directionality, and the three frequency intervals (low, medium, and high) are analysed graphically.

**Figure 17:**
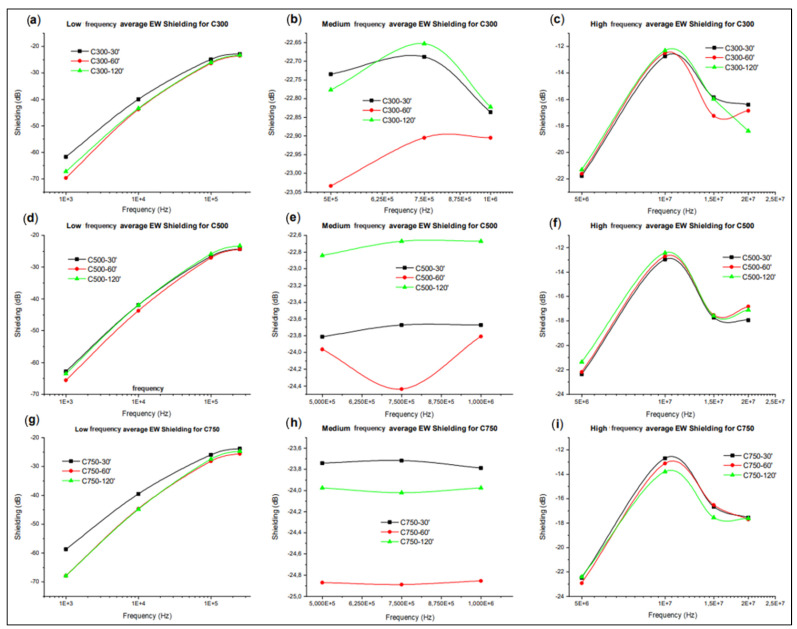
Average value of electrical signal shielding according to sonication time for systems with C300 in the low (**a**), medium (**b**), and high (**c**) frequency band; with C500 in the low (**d**), medium (**e**), and high (**f**) frequency band; and with C750 in the low (**g**), medium (**h**), and high (**i**) frequency band.

Figure 17 shows the shielding values evaluated in the three frequency ranges evaluated for each type of GNP and sonication time. From the exposed low frequency graphs for the three types of GNPs (Figure 17a,d,g) it can be concluded that the shielding behaviour according to the sonication time is the same that was found when the systems were evaluated for their DC electrical conductivities. This seems to indicate that the factors that determined the DC conductivity, such as lateral size, sonication time, amorphization, fragmentation, impurities, damage, and exfoliation, also determine the shielding of electrical signals for low frequencies. The above can be justified by stating that electrical shielding is related to AC electrical conductivity, since shielding is a measurement of the attenuation of the signal amplitude, with voltage amplitude being proportional to the resistance of the electrical signal flux through the material.

In the figures related to the medium frequency (Figure 17b,e,h) the shielding value for all types of GNP seems to indicate that the differences, and therefore the dependence of the shielding on the sonication time, is negligible. Therefore, all the factors that were affected by the sonication time, which were those that determined the behaviour of the DC electrical conductivity and the shielding at low frequencies, are no longer influential. This is related to the justification of the relationship between shielding and AC conductivity, since a frequency increment and the electrical conductivities in AC and DC are differentiated, producing a different dependence or even independence of the AC conductivity of the parameters that governed the DC conductivity.

**Figure 18:**
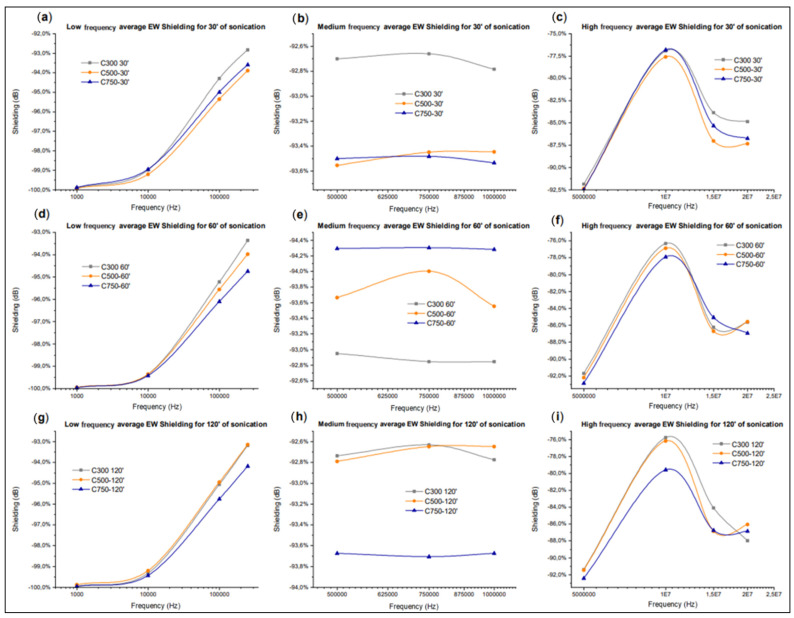
Amplitude attenuation values of the propagated electrical signal for the three types of GNP C used according to the sonication time employed and the frequency band studied. For the low (**a**,**d**,**g**), medium (**b**,**e**,**h**), and high (**c**,**f**,**i**) frequency band, the values for 30 min (**a**–**c**), 60 min (**d**–**f**), and 120 min (**g**–**i**) are shown.

Finally, in the figures related to high frequencies (Figure 17c,f,i), a recovery of the differences between the shielding values for the different sonication times is observed, but there are fluctuations that show that there is no stable behaviour for any sonication time. To conclude this section, the influence of the type of GNP used is studied (Figure 18), for which the sonication times and the frequency range are kept constant, while the directionality effect is avoided through averaging.

Figure 18 shows that C300 generates the lowest attenuations and shielding regardless of the sonication time, while C750 appears to generate the highest attenuations for sonication times of 60 and 120 min. For short sonication times of 30 min, the C500 generates the highest shielding. In addition, for longer sonication times of 60 min, above the minimum at 10 MHz, there is a trade-off of influence between the GNPs generating higher and lower shielding. It can be concluded that the type of GNP is determinant in the low and high frequency range, but not in the mid-frequency range, where it is shown to be independent. This may be related to the fact that at low frequencies the dipoles generated within the matrix enclosed between the GNPs (forming the nano or microcondensors) are more numerous the larger the surface area of the GNP used [82], so the attenuation is higher. This would indicate that the Maxwell–Wagner interfacial polarisation is influenced by the type of GNP used.

In the medium frequency range, the Maxwell–Wagner polarisation is no longer decisive, therefore the conductive shielding becomes independent of the GNP used. Finally, at high frequency it can be observed that a higher GNP surface area implies a higher shielding, probably because at these frequencies the Inductor–Capacitor (LC) behaviour of the system is determinant, as the lateral size and surface area of the GNP are influential in this behaviour [83].

## 4. Conclusions

The effects of the type of graphene on the electrical and thermal properties of the epoxy matrix nanocomposite, as well as the sonication time used for its dispersion, were studied by means of experimental techniques, justifying the properties obtained through the comparison between experimental results and literature review.

During the processing, specifically at gravity moulding stage, it has been observed that a phenomenon of self-stratification of graphene can occur, producing a concentration gradient of graphene in the Z direction, as well as unequal behaviours due to the existence of heterogeneities in the manufactured materials, such as the presence of high conductivity paths with high contents of agglomerates.

In addition, it has been shown that an increase in sonication time beyond 30 min does not allow for obtaining a homogeneous concentration of GNPs. However, an increase in the sonication time does allow for the distribution of the GNPs contained in the segregation zone, where there is a progressive increase in the fragmentation of the agglomerates and GNPs, which in turn causes a decrease in the thickness of the decanted layer. This is because the distribution of GNPs is more homogeneous, causing an increase in viscosity, which makes particle settling more difficult. This settling is also hindered by the smaller size of the GNP.

On the other hand, it was obtained that an increase in sonication time produced a loss of electrical conductivity independent of the spatial direction of measurement, and of thermal conductivity in the in-plane direction, the latter only when C300 and C500 are used, since C750 shows a larger insulating layer size with low graphene content. The Joule effect takes the same trend as the electrical conductivity with respect to the variation of the applied sonication time, and the phenomena of fragmentation, dispersion, and damage generation are the ones that compete to determine the evolution of the properties with the passage of sonication time. Regarding the analysis of the Joule effect heating, it has been shown that the existence of anisotropy in the in-plane heating strongly depends on the type of GNP used, and that there are three well differentiated stages when studying the Joule effect. This anisotropy means that for graphene-reinforced epoxy systems manufactured by gravity moulding, bias direction must be considered when performing SHM with defect locations.

A larger specific area and smaller lateral size of the GNP (C750) produces higher values of transverse electrical conductivity, lower thermal conductivities in the layering direction, and higher temperatures and heating rates. While GNPs (C300) with low specific areas and larger lateral sizes allow for reaching the highest values of electrical and thermal conductivity in the layering direction or Z direction. Those GNPs (C500) with intermediate properties, present a great homogeneity in heating by the Joule effect, which gives them the possibility of being used for SHM by infrared thermography.

The attenuation of electromagnetic conductive waves in the frequency range from 100 Hz to 20 MHz behaves independently of the heterogeneities produced by the formation of agglomerates and self-stratification on the lower surface. It is also significant that the conservation of the shielding values of the three types of GNPs are in a range of average frequencies between 0.3 and 3 MHz. The use of these materials in aeronautical applications within the inner structure of the fuselage would allow a weight reduction and the addition of a new multifunctional property, such as the attenuation of leakage currents coming from electrical systems. Exceptionally, significant differences were observed between the signal attenuation measurements between the X and Y directions, demonstrating that these electromagnetic properties maintain isotropy.

## Figures and Tables

**Table 1:** Joule effect ignition voltage for all epoxy-GnP systems. This voltage is tied to the most effective specimen within the three analysed for each system configuration. The ranges shown collect all the activation voltages of the specimens analysed for each system configuration with heating capability.

Type of GNP	Weight Percent of GNP	Sonication Time		
		30 Min	60 Min	120 Min
C300	12%	75–250 V	-	750–1000 V
C500	12%	125–250 V	500–750 V	375–500 V
C750	12%	75–250 V	400–600 V	-

**Table 2:** Exposure of mechanisms involved in electrical conduction, and therefore in the Joule effect. For each mechanism the I–V relation, the dependence of conductivity on temperature, and the necessary conditions for the intervention of each mechanism are indicated.

Conduction Mechanisms	I–V Relation Behaviour		Temperature Dependence Current	Condition of Occurrence
**Drift conduction**	I=G·V		$\left(\rho_{T}-\rho_{0}\right)=\alpha \cdot \rho_{0}\cdot \Delta T$ $\alpha =\{\begin{array}{c} +,\;\;Conductors\\ -,\;\;Insulators \end{array}\;$	Existence of a continuous path formed by GNPs, agglomerates, and dielectric breakdown. Intrinsic conduction to the GNPs or agglomerates.
**Tunnel conduction** [69]	$V<V_{act}$	$I\propto \left({∁}_{1}\right)\cdot V$	Supposed to be independent	Existence of discontinuous paths where GNPs and resin are interspersed. Tunnel effect is generated between the GNPs through the matrix.
	$V_{act}<V<V_{C}$	$I\propto \left({∁}_{2}\right)\cdot \left(V+\left({∁}_{3}\right)V^{3}\right)$		
	$V<V_{C}$	$I\propto \left(C_{4}\right)\cdot V^{2}\cdot \exp\left(-\frac{C_{5}}{V}\right)$		
**Hopping conduction** [70]	I∝V		$I\propto exp\left(-\frac{C_{6}}{T}\right)$	Above the critical voltage arises. This is because the temperature begins to be influential, activating this phenomenon.
**Cold field emission** [71,72]	$I\propto \left(C_{7}\right)\cdot V^{2}\cdot \exp\left(-\frac{C_{8}}{V}\right)$		$I\propto \;\frac{\left(C_{9}\right)\cdot T}{\sin\left(C_{9}\cdot T\right)}≅1+\frac{1}{6}$ $\cdot \left(C_{9}\right)\cdot T^{2}\approx \frac{1}{6}\cdot \left(C_{9}\right)\cdot T^{2}$	For high field strength, emission of electrons with energies below the Fermi level has high probability. Field dependence is mainly responsible for variations in the emitted current.
**Thermionic emission** [70,71]	$I\propto \left(C_{10}\right)\cdot \exp\left(\sqrt{V}-C_{11}\right)$		$I\propto \left(C_{10}\right)\cdot T^{2}\cdot \exp\left(-\frac{C_{12}}{T}\right)$	For high temperature, emission over the barrier has high probability. The temperature dependence of the distribution function is mainly responsible for variations in the emitted current.
